# Context-dependent parasite infection affects trophic niche in populations of sympatric stickleback species

**DOI:** 10.1017/S0031182022000531

**Published:** 2022-08

**Authors:** Doko-Miles J. Thorburn, Thijs M. P. Bal, Io S. Deflem, Filip A. M. Volckaert, Christophe Eizaguirre, Joost A. M. Raeymaekers

**Affiliations:** 1 School of Biological and Chemical Sciences, Queen Mary University of London, London, UK; 2 Department of Life Sciences, Imperial College London, London, UK; 3 Faculty of Biosciences and Aquaculture, Nord University, Bodø, Norway; 4 Laboratory of Biodiversity and Evolutionary Genomics, KU Leuven, Leuven, Belgium

**Keywords:** Character displacement, *Gasterosteus aculeatus*, niche specialization, parasite infection, *Pungitius pungitius*, stable isotope analysis

## Abstract

How parasites alter host feeding ecology remains elusive in natural populations. A powerful approach to investigate the link between infection and feeding ecology is quantifying unique and shared responses to parasite infection in related host species within a common environment. Here, 9 pairs of sympatric populations of the three-spined and nine-spined stickleback fishes were sampled across a range of freshwater and brackish habitats to investigate how parasites alter host feeding ecology: (i) biotic and abiotic determinants of parasite community composition, and (ii) to what extent parasite infection correlates with trophic niche specialization of the 2 species, using stable isotope analyses (*δ*15N and *δ*13C). It was determined that parasite community composition and host parasite load varied among sites and species and were correlated with dissolved oxygen. It was also observed that the digenean *Cyathocotyle* sp.'s abundance, a common directly infecting parasite with a complex life cycle, correlated with host *δ*^13^C in a fish species-specific manner. In 6 sites, correlations were found between parasite abundance and their hosts' feeding ecology. These effects were location-specific and occasionally host species or host size-specific. Overall, the results suggest a relationship between parasite infection and host trophic niche which may be an important and largely overlooked ecological factor. The population specificity and variation in parasite communities also suggest this effect is multifarious and context-dependent.

## Graphical abstract

**Figure:**
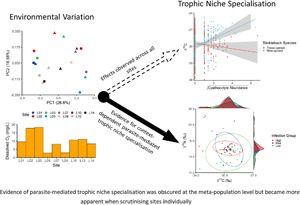

## Introduction

Parasites are strong selective agents on their hosts, reducing their fitness as well as altering their behaviour, life-history traits and habitat use (Milinski, 1984; Miura *et al*., 2006; Pagán *et al*., 2008; Lefèvre *et al*., 2009; Barber and Huntingford, 1995). Parasites are involved in approximately 75% of food web links (Lafferty *et al*., 2006), and often exceed total biomass of top predators (Kuris *et al*., 2008). Despite growing evidence that parasites have a strong impact on food web ecology (Lafferty *et al*., 2006; Dobson *et al*., 2009; Anaya-Rojas *et al*., 2019), their role in altering an individual hosts' trophic niche specialization remains largely unknown (Araújo *et al*., 2011; Pegg *et al*., 2015*a*, 2017; Britton and Andreou, 2016; Lockley *et al*., 2020). Such parasite-mediated effects on trophic niche specialization are unresolved because (i) the effects of parasites on altering host trophic niche may be system-specific, or (ii) parasites with different life-history strategies may alter trophic interactions in different ways. For example, trophically transmitted parasites often have complex life cycles and can manipulate host behaviour, therein modifying trophic interactions (Lafferty *et al*., 2008; Hammerschmidt *et al*., 2009). Specifically, through infection and regulation in all trophic levels, including top predators, parasites can induce or inhibit trophic cascades affecting overall ecosystem functioning (Brunner *et al*., 2017; Anaya-Rojas *et al*., 2019). It should be noted that hosts are not defenceless, but mounting an immunological response can be energetically costly, and therefore an appropriate feeding strategy is necessary to compensate the costs of infection (Lee *et al*., 2006; Brunner *et al*., 2014, 2017).

The theoretical effects of parasite infection on trophic niche specialization have been summarized into 2 main effects: constriction and divergence (Britton and Andreou, 2016). Trophic niche constriction occurs when a population subgroup specializes on a restricted breadth of food items that are also consumed by conspecific generalists. Indeed, parasite infection can result in the sub-population of infected hosts being nested within the overall host's population niche (Britton and Andreou, 2016; Pegg *et al*., 2017; Villalobos *et al*., 2019). For example, when comparing the trophic niche of the generalist common roach (*Rutilus rutilus*) and common bream (*Abramis brama*) infected by *Ergasilius briani*, infected individuals showed a smaller niche width that is nested within that of uninfected conspecifics (Pegg *et al*., 2017). Conversely, the overall population niche might expand due to a sub-population of infected individuals shifting diet to food items not previously exploited by conspecifics, resulting in trophic niche divergence (Britton and Andreou, 2016). Competitive exclusion of infected individuals would manifest as an overall population niche expansion. For instance, common carp (*Cyprinus carpio*) infected with the non-native Asian tapeworm (*Bothriocephalus acheilognathi*) can become competitively disadvantaged upon infection, and alter their diet compared to uninfected conspecifics (Britton *et al*., 2011; Pegg *et al*., 2015*a*).

Identifying whether parasite-induced niche constriction or divergence are a function of environmental conditions represents a logical next step in uncovering the causal mechanisms that underlie the relationship between parasitism and host trophic ecology. Indeed, abiotic environmental conditions such as variation in physico-chemistry affect important aspects of the ecology of host–parasite interactions, including host community structure, and parasite abundance and virulence (Blanar *et al*., 2009; Karvonen *et al*., 2013; Mahmud *et al*., 2017; Deflem *et al*., 2021). For example, reductions in dissolved oxygen (DO) can induce hypoxic stress in hosts which can result in ineffective immune responses, and is associated with increased parasite diversity and infection load in fish (Lacerda *et al*., 2018; Ojwala *et al*., 2018; Abdel-Tawwab *et al*., 2019). Conversely, acidification of aquatic environments can deplete intermediate host populations as in the case in many trematode parasites and their snail hosts, and is associated with reductions in parasite richness and virulence (Lafferty and Kuris, 1999; Young and MacColl, 2017).

To disentangle the relative contributions of host-related and environmental drivers of parasite-mediated shifts in host trophic niche, studies can focus on natural systems with environmental gradients and multiple related species persisting in a common landscape. In general, if genetically and ecologically similar sympatric species respond in a similar manner, the response is likely constrained by niche availability in the environment. However, if species living in sympatry respond differently to a shared environment, the responses are likely linked to mechanisms unique to a species (Raeymaekers *et al*., 2017; Bal *et al*., 2021).

The three-spined (*Gasterosteus aculeatus* Linnaeus, 1758; Gasterosteidae) and nine-spined (*Pungitius pungitius* Linnaeus, 1758; Gasterosteidae) sticklebacks are 2 small teleost fishes, which diverged around 26 million years ago (Varadharajan *et al*., 2019). Often found living in sympatry, both species demonstrate examples of overlapping dietary preference and habitat use, similar population genetic structure and parallel phenotypic divergence (Copp and Kováč, 2003; Raeymaekers *et al*., 2017; Bal *et al*., 2021). Their macroparasites are well documented, with several parasites able to infect both species, and some that are host-specific (Kalbe *et al*., 2002; Zander, 2007; Henrich *et al*., 2013). Responses to infection in both species use a similar set of immune genes (Lenz *et al*., 2013). The shared ecological context among the species provides a good opportunity to investigate the relative roles of host species, their environment and parasite infection on trophic niche ecology.

Using natural coexisting populations of *G. aculeatus* and *P. pungitius* across a range of habitats in Belgium and the Netherlands, we investigated the role of parasites in altering the trophic niche of host species, including (i) quantifying parasite diversity and abundance among host populations and their relationship with abiotic environmental variables, (ii) using stable isotope analysis (SIA; *δ*^15^N and *δ*^13^C) to assess the size of host trophic niche and investigate its relationship with parasite infection and (iii) determine whether these effects are consistent among species and sampling site, or more context-dependent. These aims test our hypotheses that infected individuals will have constricted or divergent trophic niches compared with uninfected conspecifics, and whether constriction or divergence will be linked to additional environmental variables.

## Materials and methods

### Sample and data collection

**Fig. 1.**
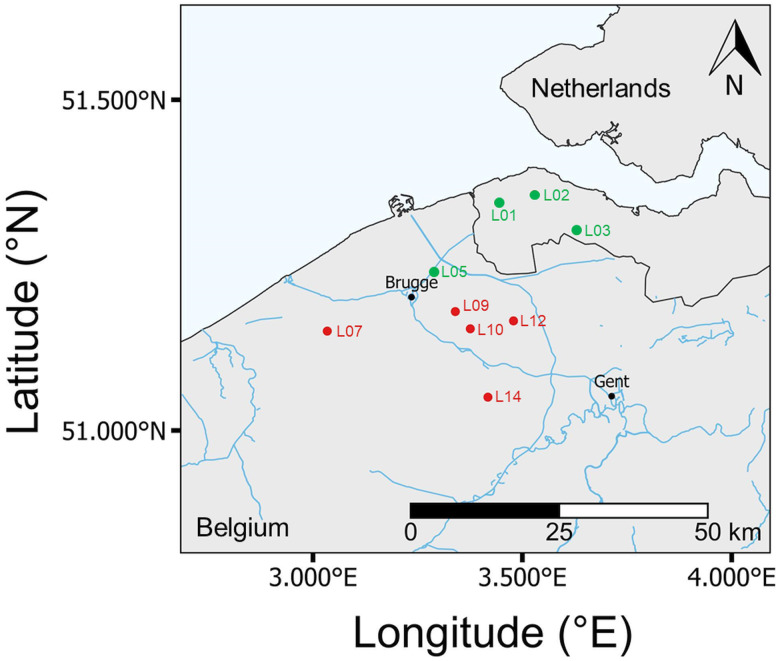
Map of study area. A total of 9 sites were sampled across a salinity gradient in Belgium and the Netherlands. Sites in green are considered brackish (conductivity ⩾ 1000 *μ*S cm^−1^), and sites highlighted in red are freshwater. Map was produced using QGIS (QGIS.org, 2021).

The coastal lowlands of the Netherlands and Belgium contain both freshwater and brackish habitats. These habitats are often dominated by 2 coexisting and phylogenetically related fish species: the 3-spined (*G. aculeatus*) and the 9-spined (*P. pungitius*) sticklebacks. Five freshwater and 4 brackish sites were sampled in March–April 2018 using dipnets (Fig. 1). Sites were selected to represent a range of salinity and were only sampled if the target species were identified living in sympatry. Sampling started with recording conductivity (*μ*S cm^−1^), pH, temperature (°C), DO (mg L^−1^), turbidity and average water depth of the site. Fish densities were then recorded across a 100 m transect using a standardized approach of dipnetting once per meter. A total of 30 individuals per species were taken from the Belgian sites, and 25 individuals per species from the Dutch sites. In the laboratory, fish from each site and species were kept separately for up to 48 h in aerated aquaria. Lighting was controlled under a 12:12 day:night regime. The fish were euthanized with a lethal dose of tricaine methanesulphonate (MS-222) and weight (g ± 0.001), length (cm ± 0.1 cm) and sex were recorded. A detailed evaluation of parasite infection was conducted for each individual, detecting the presence of parasites using a protocol developed for sticklebacks (Kalbe *et al*., 2002). First, ectoparasites were identified scanning the entire body using a stereomicroscope. Then, endoparasites were identified using a microscope and high-pressure compressorium containing the liver, gut, intestines, swim bladder, body and head, kidneys, gill arches and right eye. Finally, a muscle sample without skin was removed from the right flank of each individual, and frozen for SIA. The muscle was chosen due to the lower stable isotope turnover rate when compared with other tissues such as the liver (Guelinckx *et al*., 2008).

### Stable isotope analysis

The energy flow among trophic links can be estimated through the use of stable isotopes. In particular, nitrogen stable isotopes (*δ*^15^N) are typically enriched by 3–4‰ between consumers and their prey (Post, 2002). Carbon stable isotopes (*δ*^13^C) on the other hand inform on the initial source of carbon (i.e. littoral *vs* pelagic), enabling inferences on the feeding ecology of organisms (Post, 2002). Hence, with SIA it is possible to determine the trophic niche of individuals within and among populations and explore the link between trophic niche and parasite infection. Specifically, the study of stable isotopes and parasite infection in populations of sympatric host species can reveal whether shared parasite infections consistently alter a host's trophic niche. Here, muscle samples were dried at 60°C for 48 h, ground into a fine powder using a ball mill (Retsch UK Ltd., Hope, UK), and weighed into tin capsules (~0.8 mg; Elemental Microanalysis Ltd., Oakhampton, UK) using an ultra-microbalance (±0.001 mg; Sartorius Lab Instruments, Göttingen, Germany). Isotope ratios were analysed using continuous flow isotope mass spectrometry (Sercon, Crewe, UK). Isotope ratios are expressed in per mil (‰) relative to known reference materials for both carbon (*δ*^13^C) and nitrogen (*δ*^15^N). The C:N ratio is a proxy of body composition with respect to lipids, and in the data it indicates an overall low lipid content, negating the need to apply lipid correction protocols (Post *et al*., 2007).

### Parasite infection and the environment

Environmental conditions affect the distribution of parasites. Hence, the variation in parasite abundance among host populations and the relationship between parasite community composition and abiotic environmental variables was investigated. To conduct the analyses effectively, 2 datasets were created. The first dataset used square-root-transformed aggregated mean parasite abundance for each site and species (hereafter aggregated dataset), and the second was kept as an individual-based dataset which permitted site-specific inferences. The aggregated dataset was created to avoid pseudo-replication when comparing parasite community data to environmental variables, upon which a principal coordinates analysis (PCoA) was performed based on Bray–Curtis dissimilarities. Then a permutational multivariate analysis of variance (PERMANOVA) on the same Bray–Curtis dissimilarity matrix was performed to assess the effect of site, host species and the interaction between host species and site using the *adonis* function in the R package vegan v.2.5.5. A *post hoc* test was run using the *pairwise.adonis* function (pairwiseAdonis package v.0.4) to identify all significant pairwise combinations (Martinez Arbizu, 2020). Next, a similarity percentages test based on permutations (SIMPER) was used to identify which parasites contributed most to the differences between species and site. Finally, differences were correlated among environmental conditions (i.e. DO, pH, temperature, conductivity and total stickleback density), and the parasite community that used the PCoA axes' scores as response variables in linear models.

### Linking parasite infection and trophic niche

An index of parasite load, the Individual Parasite Index (IPI; Kalbe *et al*., 2002), was calculated for each individual fish comparing intra-individual parasite abundance to abundances. Two datasets were created. First, all samples were pooled and IPI was calculated across all sites (IPI_All_), which enables comparison of infection load across fish from all sites. However, to detect patterns of trophic niche specialization across the entire dataset, the impact of parasite infections needs to be consistent and strong. Differences in parasite community, coevolutionary histories and environmental heterogeneity all decrease the likelihood of detecting such patterns in natural systems. Hence, the second dataset was created by calculating IPI within each site separately (IPI_Site_), permitting the investigation of site-specific patterns of parasite infection that would be obscured using the whole dataset. This is needed as IPI considers variation in parasite count in the total sample.

To link parasite infection and trophic niche, the stable isotopes *δ*^15^N and *δ*^13^C of both species were used to investigate the hypothesis that parasite infection affects host trophic niche specialization. Firstly, collinearity was removed from IPI_All_ and fish length by taking the residuals of a linear model between those 2 factors. Models were then created using *δ*^15^N as the response variable, an interaction between IPI_All_, stickleback species and fish length as the fixed factor, and sampling site as a random factor. To permit comparisons among fish sizes, we grouped fish by species and size evenly into 3 groups based on length for each site. The same model was created but changing the response variable to *δ*^13^C. Stepwise model selections were performed based on the AIC criterion using the *step* function in the lmerTest package v.3.1 (Kuznetsova *et al*., 2017). In addition, previous work has shown that patterns of parasite-mediated trophic niche specialization can be achieved by a single parasite species (Pegg *et al*., 2015*a*, 2017). To investigate whether a single parasite putatively induced trophic niche specialization here, IPI_All_ was swapped for the square-root-transformed abundance of the 3 most important parasites as indicated by the SIMPER analysis (*Gyrodactylus* sp., *Neochinorhynchus* sp. and *Cyathocotyle* sp.), and Glochidia in L05 due to its extreme abundance only in this population. A minimum of 10 individual parasites from each of the 3 most common species were required to run the model.

Analyses for each site were repeated using the same model, only swapping IPI_All_ for each of the following terms individually: IPI_Site_, and the square-root-transformed abundances of *Gyrodactylus* sp., *Neochinorhynchus* sp., *Cyathocotyle* sp. and Glochidia in L05. Linear mixed-effects models were obtained with the R package lme4 v.1.1 (Bates *et al*., 2015), and *P* values were calculated using Satterthwaite's type II degree of freedom method from the lmerTest package. As each parasite load or abundance fixed factor addressed a discrete hypothesis, we corrected *P* values for multiple testing for each hypothesis. For instance, does *δ*^15^N and *δ*^13^C differ among *Cyathocotyle* sp. infections groups? As a result, each hypothesis had a maximum of 18 linear models, and *P* values were corrected for multiple testing accordingly using false discovery rate (FDR). However, for Glochidia, only 1 site (L05) had extreme abundances, so *P* values were not corrected when investigating these patterns.

To test whether parasite infection can specifically mediate host trophic niche constriction or divergence, for each site, fish were grouped by species, and then by parasite infection categories (low, medium and high). The infection categories were created by grouping individuals from each species evenly into 3 groups based upon IPI_Site_. The groupings were repeated for each of the 3 most important parasite species indicated by the SIMPER analysis and Glochidia in site L05 due to their extreme abundance only in this site. The Kolmogorov–Smirnov test was used to determine whether the low and high infection group distributions differed among the *δ*^15^N and *δ*^13^C axes within each site. Where appropriate, *P* values were corrected for multiple testing using FDR. Given the role of stickleback species denoted by previous analyses, this analysis was repeated for all sites for each host species separately. All analyses were conducted in R v4.0.2 (R Development Core Team, 2019).

## Results

### Parasite infection and the environment

In total, 19 species of parasites were identified, with *Gyrodactylus* sp. and *Cyathocotyle* sp. species being identified in every site (Supplementary Table S1). Notably, *Proteocephalus* sp., *Neochinorhynchus* sp., *Diplostomum* sp. and *Anguillicoloides crassus* were all identified in at least 7 of 9 sites. *Raphidascaris acus*, *Schistocephalus solidus* and *Paradilepsis scolecina* were the rarest parasites in *G. aculeatus*, with the latter being found in 1 site only (Supplementary Table S1); whereas only *Schistocephalus pungitii* was restricted to *P. pungitius.* Parasite diversity significantly differed among species and site, and in general, *G. aculeatus* were infected with a more diverse assemblage of parasites [analysis of variance (ANOVA) parasite diversity, *F*_17,480_ = 16.72, *P* < 0.001; Table 1], with mean individual parasite diversity differing among species and site, ranging from 1.50 ± 0.19 (standard error; s.e.) for *P. pungitius* in site L02 to 3.61 ± 0.25 for *G. aculeatus* in site L05. Similarly, parasite load (IPI_All_) differed among sites and species, with *G. aculeatus* generally having higher infection loads than *P. pungitius* (ANOVA, *F*_17,480_ = 14.41, *P* < 0.001).

**Table 1.** Summary of parasite diversity and load by site

Site	Species	Parasite diversity	Mean individual parasite diversity ± s.e.		Mean individual parasite index ± s.e.	
L01	*G. aculeatus*	8	2.160	±0.149	2.548	±0.654
L01	*P. pungitius*	8	2.120	±0.176	1.938	±0.297
L02	*G. aculeatus*	5	1.240	±0.132	0.994	±0.443
L02	*P. pungitius*	7	1.500	±0.186	2.703	±0.806
L03	*G. aculeatus*	6	1.800	±0.152	1.363	±0.253
L03	*P. pungitius*	7	1.708	±0.185	1.310	±0.275
L05	*G. aculeatus*	11	3.613	±0.248	6.990	±0.769
L05	*P. pungitius*	9	2.867	±0.207	5.866	±0.524
L07	*G. aculeatus*	9	2.517	±0.189	2.016	±0.260
L07	*P. pungitius*	6	1.724	±0.148	0.924	±0.123
L09	*G. aculeatus*	11	2.069	±0.191	3.470	±0.600
L09	*P. pungitius*	7	2.407	±0.179	2.728	±0.641
L10	*G. aculeatus*	8	1.815	±0.192	3.611	±0.678
L10	*P. pungitius*	8	0.963	±0.164	1.719	±0.497
L12	*G. aculeatus*	8	2.000	±0.126	6.983	±1.035
L12	*P. pungitius*	7	1.933	±0.126	2.550	±0.537
L14	*G. aculeatus*	10	3.724	±0.209	8.636	±1.005
L14	*P. pungitius*	8	2.533	±0.164	2.750	±0.404

**Fig. 2.**
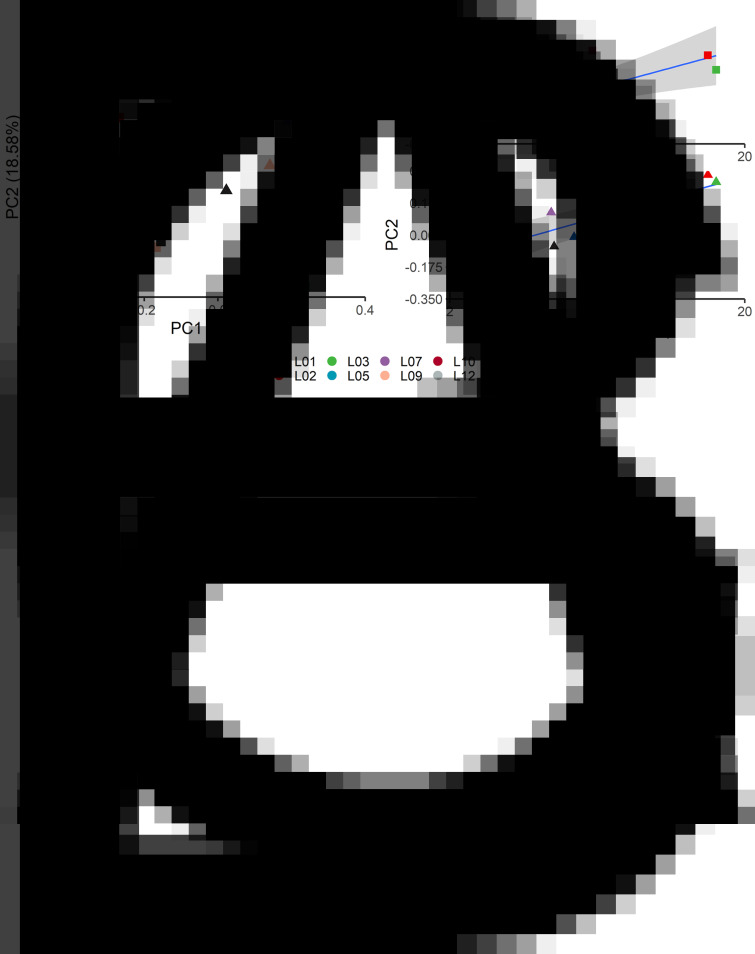
Linking parasite community composition and the environment. (A) PCoA of mean aggregated parasite communities for each sampling site and species based on Bray–Curtis dissimilarity. Figures (B–C) represent the relationships between parasite community composition (PC2) and the DO for each host (B) *G. aculeatus*, and (C) *P. pungitius*. Colours denote sampling site. Squares represent *G. aculeatus*, triangles represent *P. pungitius*. Grey shaded area represents the 95% confidence intervals.

Overall, parasite community composition significantly differed among sites and species (PERMANOVA, individual-based dataset, site:species, *F*_8,475_ = 7.36, *P* < 0.001; aggregated mean parasite abundance dataset, site, *F*_8,17_ = 3.15, *P* < 0.001, species, *F*_1,17_ = 2.76, *P* = 0.040; pairwise PERMANOVAs are reported in Supplementary Table S2; Fig. 2A). Notably, the parasite community composition among species and site, inferred from PCoA axes (PC1 and PC2), was linked to DO in both stickleback species (3S, *F*_1,7_ = 12.03, *P* = 0.010; 9S, *F*_1,7_ = 15.07, *P* = 0.006, Fig. 2B). However, when investigating whether parasite diversity or parasite load was correlated with DO, we found no significant correlations in either *G. aculeatus* (parasite diversity, *F*_1,7_ = 1.22, *P* = 0.117; IPI_All_, *F*_1,7_ = 2.60, *P* = 0.151) or *P. pungitius* (parasite diversity, *F*_1,7_ = 2.57, *P* = 0.153; IPI_All_, *F*_1,7_ = 2.51, *P* = 0.157).

Based on site by species comparisons in the individual-based dataset, SIMPER analyses revealed that the abundances of 3 parasites, *Gyrodactylus* sp., *Neochinorhynchus* sp. and *Cyathocotyle* sp., were significantly different in at least 25% of all comparisons (Supplementary Table S3). Next, using the aggregated dataset, *Diplostomum* sp. and *S. solidus* were significantly more abundant in *G. aculeatus* than in *P. pungitius* (Supplementary Table S3). Among the 2 stickleback species, both *Gyrodactylus* sp. and *Neochinorhynchus* sp. were generally more abundant in *G. aculeatus*, whereas *Cyathocotyle* sp. was more abundant in *P. pungitius* (ANOVA; *Gyrodactylus*; *F*_17,480_ = 26.80, *P* < 0.001; *Neochinorhynchus*; *F*_17,480_ = 17.17, *P* < 0.001; *Cyathocotyle*; *F*_17,480_ = 5.84, *P* < 0.001; Supplementary Table S1).

### Linking parasite infection and trophic niche

**Fig. 3.**
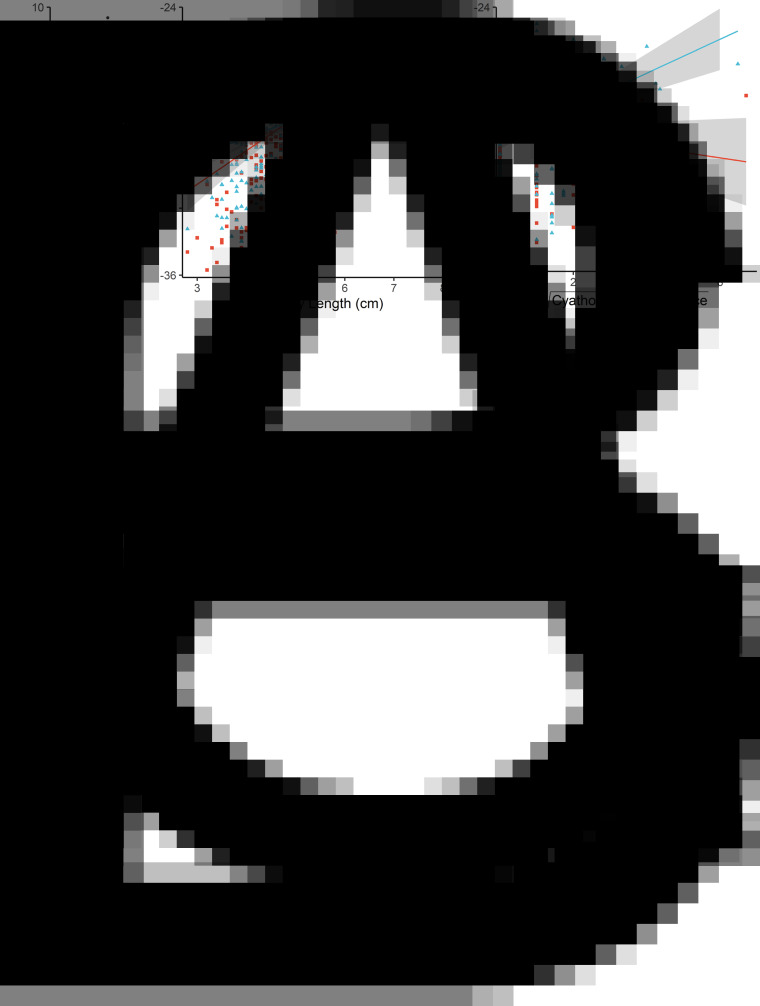
Relationship of (A) *δ*^15^N and (B–C) *δ*^13^C and their significant predictors across all sites. (A) *δ*^15^N by site residuals were plotted to remove the effect of site. (B–C) The solid lines represent the significant relationship between *δ*^13^C, stickleback species and (B) fish length or (C) *Cyathocotyle* sp. abundance. The colours represent the same species in all panels, squares represent *G. aculeatus* and triangles *P. pungitius*. Grey shaded area represents the 95% confidence intervals.

Across all sites and after model simplification, the best predictor for *δ*^15^N was stickleback species, whereby *P. pungitius* had higher *δ*^15^N mean than *G. aculeatus* (LMER, random effect variance 4.05 ± 2.01, residual variance 2.91 ± 1.70, stickleback species, *F*_488_ = 24.09, *P* < 0.001, Fig. 3A). The same stickleback species effect was detected when replacing IPI_All_ by the most common parasites, *Gyrodactylus* sp., *Neochinorhynchus* sp. and *Cyathocotyle* sp. Alternatively, *δ*^13^C was positively correlated with an interaction between fish species and size, with the slope of *G. aculeatus* being steeper than that of *P. pungitius* (LMER, random effect variance 5.73 ± 2.39, residual variance 1.24 ± 1.12, species:length, *F*_487_ = 8.48, *P* = 0.004, Fig. 3B). The same minimum adequate model was reached when replacing IPI_All_ with *Gyrodactylus* sp. or *Neochinorhynchus* sp. abundances. However, in addition to the species:length term, *δ*^13^C increased with *Cyathocotyle* abundance in *G. aculeatus*, but decreased with this parasite in *P. pungitius* (LMER, random effect variance 5.58 ± 2.36, residual variance 1.23 ± 1.11, species:length, *F*_485_ = 10.61, *P* = 0.001, *Cyathocotyle*:species, *F*_485_ = 6.90, *P* = 0.009, Fig. 3B and C).

**Fig. 4.**
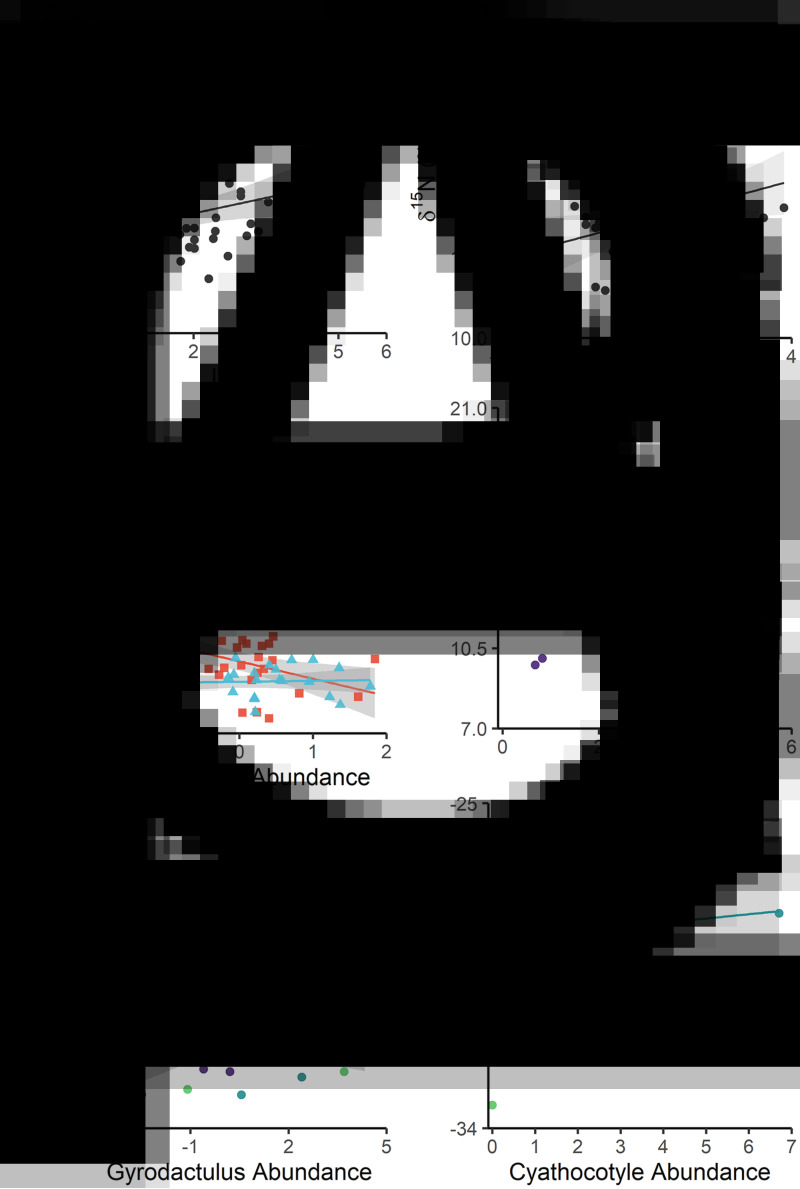
Site-specific parasite infection relationship with (A–E) *δ*^15^N and (F) *δ*^13^C. Each plot shows the significant parasite infection term in the linear mixed models (*cf.* Results and Supplementary Table S4). Figures (A–F) correspond to sampling sites L03, L05, L07, L02, L14 and L10, respectively. Circles are used when the depicted effect is across both species (A–B, D–F). When the species effect was significant, red squares were used for *G. aculeatus* and blue triangles for *P. pungitius* (D). When appropriate, to better visualize the parasite infection by host length interaction (D–F), hosts were grouped into 3 approximately equal categories: small (purple), medium (dark blue) and large (green). Where necessary (B, C, E), parasite abundances were corrected for collinearity (*cf.* Methods). Grey shaded area represents the 95% confidence intervals.

To reiterate, it was hypothesized that by mixing multiple distinct population together it would obscure any context-dependent patterns, which merited investigating sites individually. Using the site-specific parasite index (IPI_Site_), in 6 of 9 sites stickleback species was the most common predictor of both *δ*^15^N (L01, L05, L07, L14) and *δ*^13^C variation (L07, L10, L12; all models reported in Supplementary Table S4), although fish length or a species by length interaction was also significant in some sites (*δ*^15^N; L05* and L14^+^; *δ*^13^C; L12*; * indicates a species by length interaction; ^+^ indicates length as an additive effect; Supplementary Table S4). Notably, in 2 sites *δ*^15^N increases with either IPI_Site_ or the abundance of Glochidia (LM; L03, IPI_Site_, *F*_2,46_ = 4.95, FDR = 0.025; L05, Glochidia, *F*_1,56_ = 6.18, *P* = 0.016; Supplementary Table S4; Fig. 4A and B). When testing *δ*^15^N variation against *Cyathocotyle* sp. abundance, a host species and parasite abundance interaction was observed in 1 site, with the correlation for *P. pungitius* being positive and the correlation for *G. aculeatus* being negative (L07, *F*_1,54_ = 8.47, FDR < 0.001; Fig. 4C). Furthermore, stable isotope variation was explained by the interaction between parasite load (IPI_Site_) and fish length in 3 sites. Firstly, in site L02 the correlation between *δ*^15^N and IPI_Site_ was positive for large fish and negative for small fish (L02, *F*_1,45_ = 4.23, FDR = 0.009; Fig. 4D). Secondly, in site L14 the correlation between *δ*^15^N and *Gyrodactylus* sp. was negative for large fish and positive for small fish (L14, *F*_4,54_ = 7.55, FDR = 0.001; Fig. 4E). Thirdly, in site L10 the correlation between *δ*^13^C and *Cyathocotyle* sp. abundance was positive for large fish and negative for small fish (*F*_4,49_ = 5.40, FDR = 0.004, Fig. 4F).

### Trophic niche constriction and divergence

**Fig. 5.**
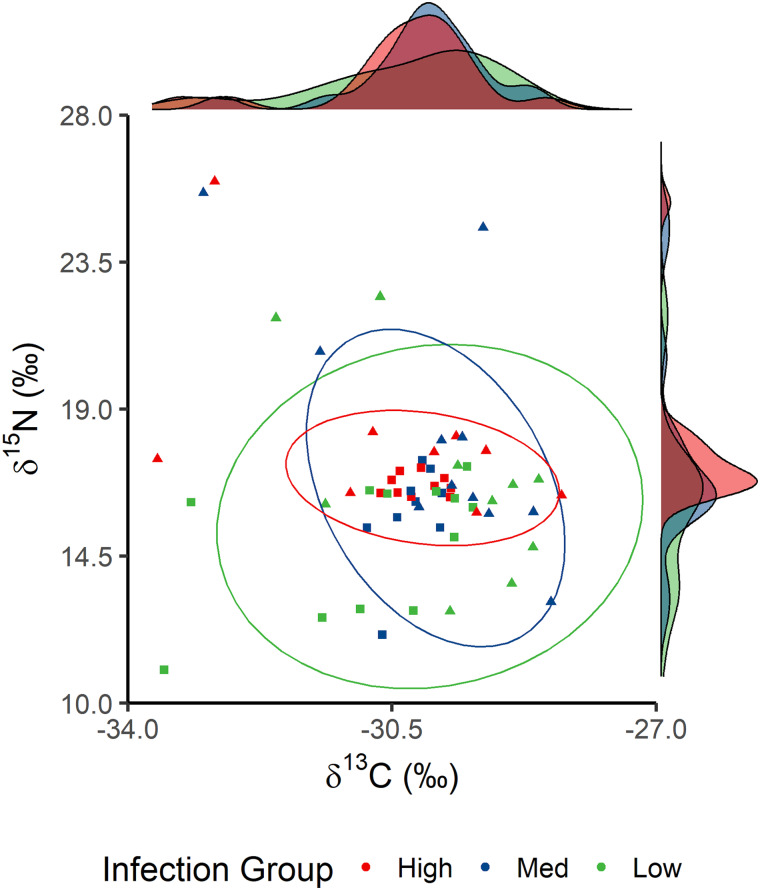
Differences in trophic niche among Glochidia infection groups in sampling site L05. Fish were separated into approximately equal groups based on Glochidia abundance. Density of each infection group is plotted along the side of each axis. Squares represent *G. aculeatus* and triangles *P. pungitius*.

There were no significant differences in trophic niche distributions when comparing low and high infection groups in each site (Supplementary Table S5, Fig. S1). However, when scrutinizing sites where specific parasites were linked to trophic niche (namely, L05*_δ_*_15N_-Glochidia, L07*_δ_*_15N_-*Cyathocotyle* sp., L10*_δ_*_13C_-*Cyathocotyle* sp. and L14*_δ_*_15N_-*Gyrodactylus* sp.), it was found that fish in site L05 with high infection of Glochidia had a significantly reduced *δ*^15^N breadth compared to fish with low infection (2-sample Kolmogorov–Smirnov test, *D* = 0.5383, *P* = 0.003; Fig. 5). All other trophic niche distributions were not significantly different (Supplementary Table S5). Yet, when considering trophic niche use as a character, evidence of character displacement among the stickleback species was observed in trophic niche axes in 6 of 9 sites (Supplementary Table S5). Specifically, constricted trophic niches with *G. aculeatus* having the broader niche were observed in 2 sites (2-sample Kolmogorov–Smirnov test; L02, *D_δ_*_15N_ = 0.408, FDR*_δ_*_15N_ = 0.045; L07, *D_δ_*_15N_ = 0.483, FDR*_δ_*_15N_ = 0.007, *D_δ_*_13C_ = 0.690, FDR*_δ_*_13C_ < 0.001; Supplementary Fig. S2) and *P. pungitius* with the broader niche also in 2 sites (L05, *D_δ_*_15N_ = 0.434, FDR*_δ_*_15N_ = 0.012; L07, *D_δ_*_13C_ = 0.469, FDR*_δ_*_13C_ = 0.007; L09, *D_δ_*_15N_ = 0.425, FDR*_δ_*_15N_ = 0.019; Supplementary Fig. S2). Finally, partial trophic niche divergence was observed in 2 sites (L10, *D_δ_*_15N_ = 0.481, FDR*_δ_*_15N_ = 0.0100, *D_δ_*_13C_ = 0.630, FDR*_δ_*_13C_ < 0.001; L14, *D_δ_*_15N_ = 0.464, FDR*_δ_*_15N_ = 0.007; Supplementary Fig. S2).

## Discussion

Focusing on 2 coexisting and phylogenetically related species and their parasites, an environmental determinant of parasite infection was identified and to what extent parasites affect trophic niche specialization was tested. Firstly, parasite community composition was correlated with DO. Secondly, parasite load and the abundance of specific parasite species were regularly negatively correlated with hosts' stable isotope values (*δ*^15^N or *δ*^13^C). These patterns were often site-specific, highlighting the context-dependent nature of parasite-mediated selection on niche use. Of the predicted patterns of parasite-mediated trophic niche specialization (Britton and Andreou, 2016), one case of trophic niche constriction was observed, where the trophic niche of the highly infected fish was entirely nested within trophic niche of the lowly infected fish. Thirdly, both species differed in trophic niche use at the majority of the study sites, albeit not consistently or in the same direction. Together, the results suggest that both host species and parasite infection simultaneously affect niche specialization and the directions of these effects are context-dependent.

### Parasite community composition and the environment

Many parasite species spend the vast majority of their lifetime, and for some species their entire lifetime, inside their hosts. Therefore, whether environmental conditions directly affect parasite abundance and diversity is still debated (e.g. Sweeny *et al*., 2021). Here, a correlation between parasite community composition and DO was observed in both species. Such correlations can independently or jointly shape host–parasite interactions (Johnson *et al*., 2007; Brunner *et al*., 2017; Ojwala *et al*., 2018). Indeed, variation in assemblages of parasites has been shown to correlate with DO in other aquatic systems (Lacerda *et al*., 2018; Ojwala *et al*., 2018; Abdel-Tawwab *et al*., 2019). However, when investigating whether specific measures of parasite community composition – namely parasite diversity and load – were also correlated with DO (and the other environmental variables: pH, temperature, conductivity and total stickleback density) we found no significant correlations. Such a result highlights the complex nature of the interaction between parasite communities and their environments over a spatial scale.

### Linking parasite infection and trophic niche

The effects of parasite infection on host physiology and behaviour are well documented (Barber and Dingemanse, 2010; Abdel-Tawwab *et al*., 2019). However, relatively little is known about the role of parasite infection on host trophic niche use in nature (Britton and Andreou, 2016; Brunner *et al*., 2017). There are a few empirical examples of an association between parasite infection and host trophic niche constriction and divergence (Pegg *et al*., 2015*a*, 2017; Villalobos *et al*., 2019), but whether environmental conditions, such as abiotic factors, interspecific competition or host density, affect these patterns remain elusive. Across the entire dataset, a correlation between *Cyathocotyle* sp. abundance and *δ*^13^C was observed, with a host-specific direction of this correlation being observed. *Cyathocotyle* sp. is a trematode parasite that uses snails as intermediate hosts before actively infecting fish hosts, including both stickleback species (Lenz *et al*., 2013; Kvach *et al*., 2016). Trematode parasites are known to affect host locomotion and trigger host immune responses (Khan *et al*., 2003; Goodman and Johnson, 2011), including in fish hosts (Barber *et al*., 2000; Binning *et al*., 2017). Such effects are consistent with species-specific shifts in *δ*^13^C, which suggest increasing *Cyathocotyle* sp. infection load is associated with change in host diet and may be a proxy or driver of displacement in niche use among host species. While both species share a similar landscape, the different responses to *Cyathocotyle* sp. infections likely stem from species-specific immune response and may be an example of parasite-mediated trophic niche divergence.

Individual trophic niche specialization within an ecosystem can be associated with differences in adaptive and behavioural traits such as body shape, body size, gape size, gill-raker morphology and habitat utilization, differentially exposing hosts to parasites (Cucherousset *et al*., 2011; Svanbäck and Schluter, 2012; Pegg *et al*., 2015*b*; Britton and Andreou, 2016; Barry *et al*., 2017). Recently, parasites have also been speculated as a driver of trophic niche specialization (Pegg *et al*., 2015*a*, 2015*b*, 2017; Britton and Andreou, 2016). Here, parasite load – specifically the abundances of *Gyrodactylus* sp., *Cyathocotyle* sp. or Glochidia – was each associated with alterations of host *δ*^15^N or *δ*^13^C. Parasite infections can lead to significant phenotypic changes in their hosts (Miura *et al*., 2006; Britton and Andreou, 2016; Anaya-Rojas *et al*., 2019). Theory also predicts infected individuals to have different optimal diets due to differences in phenotypic capacity to detect, capture and digest the available prey (Araújo *et al*., 2011). Hence, the occasionally host size- or host species-dependent nature of the observations may be a consequence of the variation in parasite assemblages and the resultant parasite induced phenotypes within and among sites. Additionally, it should be noted that previous studies identifying consistent patterns of parasite-mediated trophic niche specialization targeted non-native parasites on native fishes (Pegg *et al*., 2015*a*, 2017). In the present study, the effects of both native and non-native parasites were included, where the ecological impact of each may not be in the same magnitude or direction.

Competition within and among species in an environment is another driver of individual niche specialization (Araújo *et al*., 2011; Evangelista *et al*., 2014; Newsome *et al*., 2015). Here, host species was the strongest and most consistent determinant of trophic niche. When considering trophic niche use as a character, these results are suggestive of interspecific competition and character displacement (Schluter and McPhail, 1992; Gray and Robinson, 2002). Given the strength of species interaction, it is difficult to evaluate the role of parasite infection. As parasites contribute to host local adaptation and even to speciation (Eizaguirre *et al*., 2009; Brunner and Eizaguirre, 2016), it seems likely they also influence these evolutionary mechanisms through processes like parasite spillover and parasite-mediated phenotypic modifications (Britton and Andreou, 2016). Finally, some of the patterns observed may also be summarized by differences in adaptive potential among host species, which is underpinned by differences in demographic histories (Raeymaekers *et al*., 2017; Bal *et al*., 2021). Specifically, *G. aculeatus* tends to rapidly adapt to changing local environmental conditions while *P. pungitius* instead has the capacity to tolerate a wide range of conditions (Raeymaekers *et al*., 2017; Bal *et al*., 2021).

### Trophic niche constriction and divergence

After capturing the role of the environment and host species on parasite community and host trophic niche, the main hypotheses of this study can be tested: can parasites drive trophic niche specialization in a consistent and measurable manner (Britton and Andreou, 2016)? Here, the majority of the results showed context-dependent patterns of parasite-mediated shifts in trophic niche, if detectable at all. Notably, in 1 site (L05), parasite-mediated trophic niche constriction was observed, whereby the trophic niche of highly *Cyathocotyle* sp. infected fish was nested in that of lowly infected fish. A previous study showed similar patterns of trophic niche constriction suggesting it is likely due to infected conspecifics compensating the costs of infection by consuming specific food items already within the population's dietary breadth (Pegg *et al*., 2017). Another factor to consider is differences in dietary preferences among life stages have also been shown to differentially expose hosts to parasites (Barry *et al*., 2017). However, given the field nature of the study, we are unable to identify whether the parasitized phenotype is the result of infection or whether existing dietary preferences differently exposed subpopulations to parasites (e.g. Cucherousset *et al*., 2011; Pegg *et al*., 2015*b*; Barry *et al*., 2017). Regardless, one explanation for this observation is that parasite-mediated trophic niche constriction is driven by host diet compensation, and matches the only other known study to identify such a pattern (Pegg *et al*., 2017). Overall, such findings highlight the need for further investigations into the causal mechanisms underlying this ecological phenomenon.

## Conclusions

Overall, research on the determinants of trophic niche specialization has primarily identified factors such as competition, predation and resource availability as leading causes (Araújo *et al*., 2011). Whilst the results principally contribute to evidence supporting the impact of interspecific competition, the role of parasitism in trophic niche specialization remains poorly resolved (Britton and Andreou, 2016). Hence, studies observing an instance of trophic niche constriction, and multiple observations of parasitism putatively affecting the trophic niche of hosts are important. Such results suggest that consequences of parasite infection are an important driver of niche specialization which have been largely overlooked.

## Supplementary material

For supplementary material accompanying this paper visit https://doi.org/10.1017/S0031182022000531. click here to view supplementary material
