# Supplementary material for: Context-dependent parasite infection affects trophic niche in populations of sympatric stickleback species
Source: Parasitology. 2022 May 16;149(9):1164–72. doi: 10.1017/S0031182022000531 (PMC10090597; doi:10.1017/S0031182022000531)

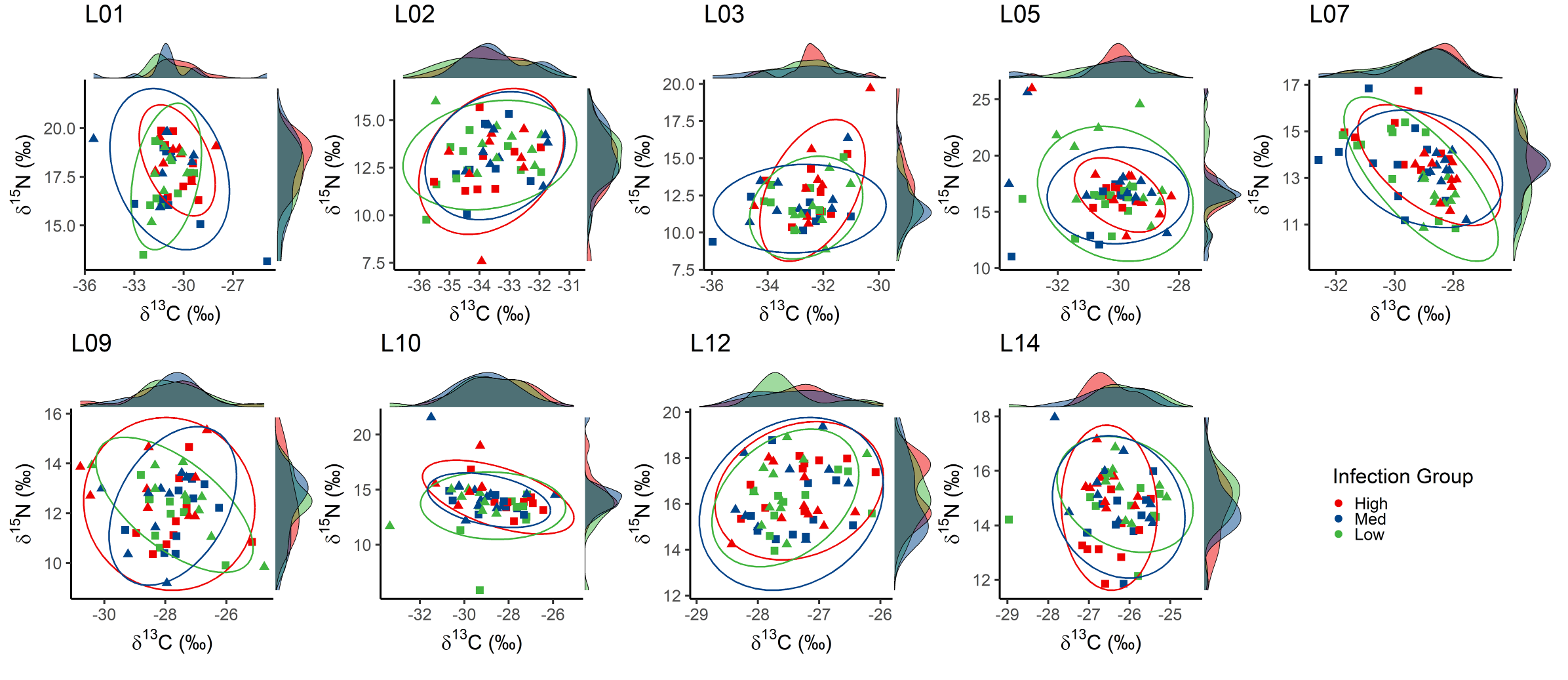
**Supplementary Figure S1. Trophic nice among IPI_Site_ infection groups.** For each site, fish were separated into approximately equal groups based on site-specific infection load (IPI_Site_). Density of each infection group is plotted along the side of each axis. Site number is indicated in the top left of each plot facet.

**Supplementary Figure S2. Trophic nice among species.** For each site, fish were separated by species. Density of each species is plotted along the side of each axis. Site number is indicated in the top left of each plot facet.


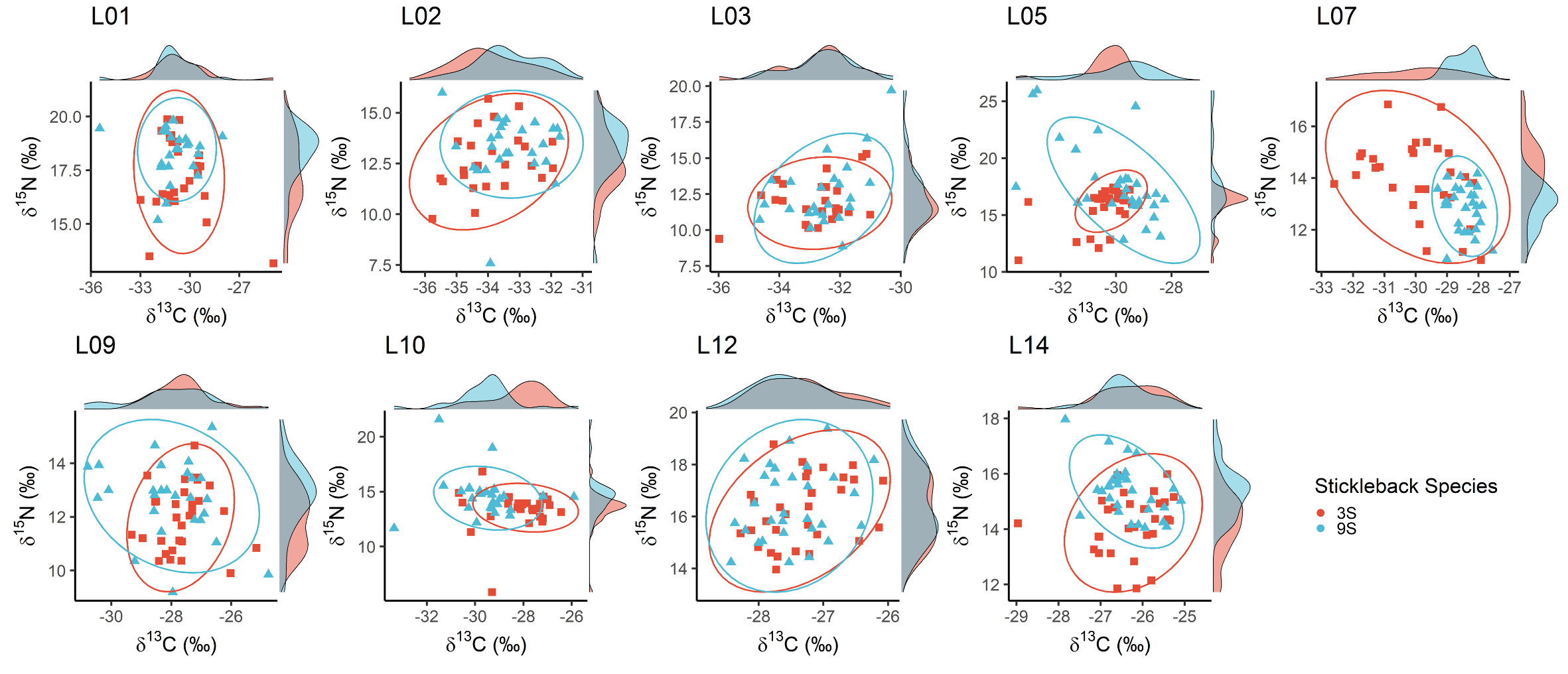

Supplement: Supplementary file 1 [file S0031182022000531sup.zip › S0031182022000531sup002.docx]
